# Antimicrobial susceptibility of uropathogens isolated from Cambodian children

**DOI:** 10.1179/2046905515Y.0000000008

**Published:** 2016-04-27

**Authors:** Catrin E. Moore, Soeng Sona, Sar Poda, Hor Putchhat, Varun Kumar, Sun Sopheary, Nicole Stoesser, Rachel Bousfield, Nicholas Day, Christopher M. Parry

**Affiliations:** ^a^ Mahidol-Oxford Tropical Medicine Research Unit (MORU), Faculty of Tropical Medicine, Mahidol University, Bangkok, Thailand; ^b^ Centre for Tropical Medicine and Global Health, Nuffield Department of Medicine, University of Oxford, Churchill Hospital, Oxford, UK; ^c^ Angkor Hospital for Children, Siem Reap, Cambodia; ^d^ Clinical Sciences, Liverpool School of Tropical Medicine, UK

**Keywords:** Urinary tract infection, Cambodia, Children; *Escherichia coli*, ESBL, Resistance

## Abstract

Background: Bacterial resistance to commonly used antimicrobials is an increasing problem in Asia but information concerning the antimicrobial susceptibility of bacteria causing urinary tract infections (UTIs) in children is limited.

Methods: This was a 5-year retrospective study of children with suspected UTI attending a paediatric hospital in north-west Cambodia. Urines with a positive culture containing a single organism with a count of >10^5^ colony-forming units (CFU)/ml were considered diagnostic of infection. The organism was identified and the resistance pattern (using CLSI guidelines) and presence of an extended-spectrum β-lactamase (ESBL) phenotype was determined.

Results: In total, there were 217 episodes of infection, 210 (97%) with Gram-negative bacteria. *Escherichia coli* was the most common infecting isolate with high levels of resistance to most oral antibiotics, except nitrofurantoin. Nearly half of the *E. coli* (44%) were extended-spectrum cephalosporin (ESC)-resistant with the proportion increasing significantly over the 5-year period. ESC-resistant *E. coli* were more likely to be multi-drug-resistant and 91% demonstrated an ESBL phenotype.

Conclusion: The data highlight the importance of microbiological surveillance of UTIs in children, particularly in areas where there are known to be multiply resistant organisms.

## Introduction

Urinary tract infections (UTI) are common in children, and as many as 2% of children have been diagnosed with at least one UTI by the age of 10 years.^1^ Prompt and appropriate antimicrobial therapy should lead to rapid recovery and the avoidance of complications. Delayed or incorrect antibiotic treatment may result in recurrence, particularly in children with renal abnormalities who risk developing progressive renal damage with hypertension and chronic renal failure.^2^ ^,^ ^3^ Vesico-ureteric reflux (VUR) is the most common underlying urinary tract disorder associated with UTI in children.^4^

Antibiotic choice should be based on local circulating bacterial strains and resistance profiles, which vary between countries. In a number of epidemiological surveys, the commonest aetiological cause of UTI in children has been *Escherichia coli*.^5^ ^,^ ^6^ Increasing levels of infection with extended-spectrum β-lactamase (ESBL)-producing *E. coli* strains have been described in Phnom Penh, the capital of Cambodia, and neighbouring countries.^7^ ^–^ ^9^ We report the species and antimicrobial susceptibility of bacteria causing UTIs in children attending a paediatric hospital in north-west Cambodia.

## Methods

### Patients

This was a retrospective analysis of laboratory data routinely collected by the Microbiology Department in Angkor Hospital for Children (AHC), Siem Reap town, north-west Cambodia. This charitable hospital provides medical care to Cambodian children ( < 16 years) free of charge and has 70 beds with approximately 125,000 attendances and 4000 admissions per year. Urine samples submitted to the laboratory between 1 January 2007 and 31 December 2011 were studied. Because of the association between UTI and bacteraemia,^10^ the hospital database was examined to discover whether any patients with UTI infections had concomitant bacteraemia.

### Laboratory methods

Urine samples (mid-stream clean-catch samples for older children and a urine bag for younger children and infants) were examined by microscopy. Samples containing >10 white blood cells/ml were cultured semi-quantitatively on Oxoid *Brilliance* UTI clarity agar (Oxoid, Basingstoke, UK) and incubated aerobically at 37°C for 18–24 hours. Cultures of a single organism with a count of >10^5^ colony-forming units (CFU)/ml were considered to represent infection and were identified using appropriate routine identification methods including Gram-stain, an in-house short set of biochemical tests and a commercial biochemical analytical profile index kit (API, BioMérieux, France). Routine diagnostic antimicrobial susceptibility results were determined using the disk diffusion method for ampicillin, co-amoxiclav, co-trimoxazole, ciprofloxacin and gentamicin in accordance with the Clinical and Laboratory Standards Institute guidelines (CLSI),^11^ and data for these routine tests were retrieved from laboratory records. Repeat, confirmatory, susceptibility testing for these antimicrobials and for species identification and additional susceptibility testing were performed on fresh sub-cultures of frozen, clinical isolates which had been stored at − 80°C for quality assurance and to minimise differences caused by changes in interpretive guidelines over the 5-year period. Additional susceptibility tests were undertaken for nitrofurantoin, mecillinam, fosfomycin, chloramphenicol, imipenem and amikacin (the latter for gentamicin-resistant isolates only). *E. coli* and Klebsiella species resistant to extended-spectrum cephalosporins (ESC, ceftriaxone, cefpodoxime or ceftazidime) were examined for ESBL/AmpC beta-lactamase (AmpC) activity in accordance with CLSI guidelines.

Repeat isolates cultured from the same patient were considered to be duplicate isolates representing a single infection if isolated within a 3-month period. The age and gender of the child and year of isolation was also recorded.

### Data analysis

Data were analysed using Stata 13·1 (StataCorp, TX, USA). Fishers Exact and χ^2^ tests were performed to examine the difference in antimicrobial susceptibilities over time, and *P* < 0·05 was taken as significance.

### Ethics approval

Ethics approval of the study was granted by the AHC Institutional Review Board, Siem Reap, Cambodia and the Oxford Tropical Research Ethics Committee, UK.

## Results

During the study period, 28,732 samples were processed by the laboratory (>5000/year) and approximately 800 samples were cultured each year. In 2011, for example, 725 urines were cultured: there was no growth in 39·7% (288), growth of < 10^4^ CFU/ml in 35·9% (260), mixed growth of more than two organisms in 10·6% (77), pure growth of 10^4^–10^5^ CFU/ml in 1·9% (13), and pure growth of a single isolate at >10^5^ CFU/ml in 11·6% (84). Over the study period, 226 single isolates were retrieved. Nine strains were considered to be duplicates, leaving 217 episodes of infection for analysis during the 5-year period. Age and gender were known for 213 patients: 66·2% (141) were female and the median age (inter-quartile range, range) was 1·73 years (10 mths to 6·2 yrs, 13 days to 16 yrs). There were 161 (74%) samples submitted from children in the outpatient department and 56 (26%) from in-patients. A blood culture was taken at the same time in 76 (35%) children with a UTI: three blood samples were positive for the same organism (*Klebsiella pneumoniae* in two and *E. coli* in one).

### Organisms

There were Gram-negative bacteria in 97% (210/217) of isolates with *E. coli* being the most common organism (170/217 isolates, 78%). Other identifiable Gram-negative isolates were *K. pneumoniae* (8·8%), *Proteus mirabilis* (2%), Pseudomonas (2%), Acinetobacter species (2%), and *Burkholderia cepacia*, *Citrobacter freundii* and Serratia species (one isolate each). All isolated Gram-positive organisms were Enterococcus species (3%); no staphylococci were isolated.

### Antimicrobial susceptibility patterns

The antimicrobial susceptibilities of the most common organisms, *E. coli* and *K. pneumoniae*, are shown in Table 1. Resistance to most antibiotics tested was common in both organisms. *E. coli* resistance to ampicillin was present in 96% (164/170) whereas *K. pneumoniae* are intrinsically resistant to ampicillin. For *E. coli* and *K. pneumoniae* combined, co-amoxiclav resistance was present in 81% (153/189), co-trimoxazole in 87% (165/189), ciprofloxacin in 47% (89/189), gentamicin in 49% (92/189) and ESC in 44% (84/189). Only 7% (12/188) of strains were resistant to nitrofurantoin. Statistically significant differences in resistance prevalence between *E. coli* and *K. pneumoniae* were observed for gentamicin (45% *E. coli*-resistant *vs* 79% *K. pneumoniae*, *P* = 0·005), ESC (40% *vs* 84%, *P* = 0·005) and nitrofurantoin (4% *vs* 26%, *P* < 0·001).

**Table 1:** Antimicrobial susceptibilities for urinary *Escherichia coli* and *Klebsiella pneumoniae* by year of isolation

	*E. coli*, *n* (%) susceptible by year							*K. pneumoniae*, *n* (%) susceptible by year*					
	2007	2008	2009	2010	2011	Total		2007	2008	2009	2010	Total	
	*n = *37	*n = *42	*n = *40	*n = *28	*n = *23	*n = *170	*P-value*	*n = *6	*n = *6	*n = *4	*n = *3	*n = *19	*P-value*
Ampicillin	2 (5)	1 (2)	2 (5)	1 (4)	0	6 (4)	0·80	-	-	-	-	-	-
Co-amoxiclav	14 (38)	6 (14)	10 (25)	2 (7)	2 (9)	34 (20)	0·02	1 (17)	0	0	1 (33)	2 (11)	0·51
Co-trimoxazole	5 (14)	5 (12)	6 (15)	5 (19)	2 (9)	23 (14)	0·90	0	1 (17)	0	0	1 (5)	1·00
Nitrofurantoin	37 (100)	39 (93)	38 (97)	28 (100)	21 (91)	163‡ (96)	0·21	4 (67)	4 (67)	4 (100)	2 (67)	14 (74)	0·74
Ciprofloxacin	26 (70)	22 (52)	19 (48)	17 (61)	9 (39)	93 (55)	0·13	3 (50)	2 (33)	0	2 (67)	7 (37)	0·30
Gentamicin	20 (54)	23 (55)	22 (45)	16 (57)	13 (57)	94 (55)	1·00	2 (33)	1 (17)	0	1 (33)	4 (21)	0·70
ESC‡	31 (84)	22 (52)	23 (58)	16 (57)	10 (43)	102 (60)	0·01	2 (33)	0	0	1 (33)	3 (16)	0·28

*P-*value derived from the logistic regression assessing the significance of changes in susceptibility over time;

* No *K. pneumoniae* isolated in 2011; ^†^ only 169 isolates tested;

‡ extended-spectrum cephalosporin

The proportion of *E. coli* strains resistant to ESC increased significantly over the 5-year period (overall *P* = 0·01), but this was not the case for *K. pneumoniae* (*P* = 0·34). Of the 68 *E. coli* strains resistant to ESC tested, 62 (91%) had an ESBL phenotype and 19 (28%) were resistant to cefoxitin, suggesting the presence of an AmpC enzyme; 13 (19%) strains were both ESBL positive and resistant to cefoxitin. *E. coli* strains with ESC resistance were significantly more likely to be also resistant to ampicillin (*P* = 0·04), co-amoxiclav (*P* < 0·001), ciprofloxacin (*P* < 0·001) and gentamicin (*P* < 0·001) than those which were ESC-susceptible (Table 2). The majority of *E. coli* strains were susceptible to mecillinam (135/169, 80%), fosfomycin (168/169, 99%), amikacin (76/76, 100% of those resistant to gentamicin), nitrofurantoin (163/169, 96%), chloramphenicol (90/169, 53%) and imipenem (169/169, 100%).

**Table 2:** Comparison of the antimicrobial susceptibilities of *Escherichia coli* isolates according to their susceptibility to extended-spectrum cephalosporins

		Extended-spectrum cephalosporin (ESC) result		
Antibiotic susceptibility	Total no. susceptible *n = *170 (% of tested)	Susceptible *n = *102 (60%)	Resistant *n = *68 (40%)	*P-value*
Ampicillin	6 (4)	6 (5·9)	0	0·04
Co-amoxiclav	102 (60)	34 (33)	0	< 0·001
Co-trimoxazole	23 (14)	18 (18)	5 (7)	0·06
Nitrofurantoin	163 (96)	99* (98)	64 (94)	0·18
Ciprofloxacin	93 (55)	77 (75)	16 (24)	< 0·001
Gentamicin	94 (55)	73 (72)	21 (31)	< 0·001
Mecillinam	135 (81)	79* (78)	56 (82)	0·51
Imipenem	169† (100)	101* (100)	68 (100)	NT (all susceptible)
Fosfomycin	168† (99)	100* (99)	68 (100)	0·41
Amikacin‡	76† (100)	29* (100)	47 (100)	NT (all susceptible)
Chloramphenicol	90†(53)	57*(56)	33 (49)	0·31

* The one strain unable to be resuscitated was ESC-susceptible and therefore only 101 ESC-susceptible strains could be analysed for these antibiotics;

† 169 samples tested, one strain could not be resuscitated for further testing;

‡ amikacin was tested only on strains resistant to gentamicin (*n = *76); NT, not tested

## Discussion

This study has demonstrated very high levels of resistance to antibiotics in *E. coli* isolates causing UTIs in Cambodian children. Less than one quarter of isolates were susceptible to oral antibiotics such as amoxicilin, co-amoxiclav and co-trimoxazole which are commonly used to treat UTIs in local children. The high levels of resistance to ESC means that oral cephalosporins would also be unsuitable, and the high levels of ciprofloxacin resistance rule it out as a reliably effective second-line choice. Susceptibility to nitrofurantoin, fosfomycin and mecillinam was examined, although they are not commonly used locally. Although nitrofurantoin is affordable ($8 for 100 tablets) and available in Cambodia, it is not routinely used, but fosfomycin and mecillinam are not currently available to purchase in Cambodia. Resistance was < 1% for fosfomycin and 18–22% for mecillinam, suggesting that fosfomycin may be effective in the current setting. In a study conducted in the capital, Phnom Penh, resistance to fosfomycin was observed in adults.^13^ The resistance to mecillinam, not seen in other studies, suggests that this antibiotic should be used with caution in our setting.^14^ ^–^ ^16^ For children with urosepsis, gentamicin, ESC and ciprofloxacin would not be reliable empirical choices. The carbapenems and amikacin would be more predictably active, but these are not widely available in most local health-care settings. The data from this study has been disseminated to physicians working at AHC and has contributed to the development of the hospital's antimicrobial guidelines.

The rates of resistance were consistent with data from an earlier study in the Cambodian capital (2004–2005) performed mainly in adults.^12^ Susceptibility to the ESC and co-amoxiclav decreased significantly over the 5-year study period. Many of the resistant strains demonstrated an ESBL phenotype although the exact enzymes involved have not been determined; in earlier published data these were shown to mostly contain CTX-M-14 or CTX-M-27 variants.^12^ Isolates with ESC were also commonly resistant to gentamicin and ciprofloxacin.

The study has a number of limitations. It is likely that many of these children had already been pre-treated with antibiotics in the community but had not responded, given that in Cambodia antibiotics are available over the counter and without prescription.^12^ Information about prior treatment was not routinely documented and might be unreliable. In a recent study, antibiotic activity could be detected in the urine of one third of children attending the AHC outpatient department.^17^ Prior treatment is also likely among children admitted to hospital. This might have resulted in overestimating the real prevalence of resistance in community-associated UTIs. The data were collected retrospectively and it is possible that some patients presenting with UTI symptoms might not have been registered on the electronic database and were missed. Furthermore, in the absence of relevant clinical data it is not certain that all episodes represented true infections. Additional data such as malnutrition, known to be prevalent in Cambodian children,^18^ were not available for the current study. Prospective studies of urinary tract disorders in children would allow collection of more comprehensive clinical and demographic information.

It is difficult to obtain a clean-catch urine sample from young children, and a number of children with genuine infections, but with a mixed growth cultured from urine, may have been misclassified. The presence of white cells in the urine and significant growth (>10^5^ CFU/mL) of a single organism is widely used as a diagnostic measure by which to identify the majority of significant infections. There was only a handful of *K. pneumoniae* isolates, and therefore limited power to detect any significant trends in resistance. Three children had a bacteraemia diagnosed at the same time as the UTI but these data may be limited if urine samples were not sent from all septic patients on admission to the hospital.

The data emphasise the value of microbiological examination of urine in children who have not responded to an initial course of antibiotics, and suggest the circulation of multiply resistant *E. coli* isolates in this setting. The high levels of resistance are consistent with the worldwide increase in the incidence of disease caused by resistant organisms, most notably ESBL-producing organisms.^19^ It is likely that multiple factors are contributing to this increase, including the easy availability of over-the-counter antimicrobials in countries in south-east Asia such as Cambodia, the inadequate quality of prescriptions dispensed and the variable quality of the drugs.^20^ The lack of new antimicrobials means that therapeutic options for many common infections such as UTI are narrowing. The work highlights the critical importance of pathogen and resistance surveillance over time.

## Disclaimer Statements

The study sponsors had no role in the study design, the collection, analysis, or interpretation of the data, the writing of the report, or the decision to submit the paper for publication.

## Contributors

CEM, SS, SR, HP performed laboratory testing. VK, SS, NS, RB, CMP researched patient databases CEM, ND, CMP edited the first draft of the paper with all authors contributing to further drafts.

## Funding

The microbiology service at AHC is part-funded by the Wellcome Trust of Great Britain, London, UK and the Li Ka Shing-University of Oxford Global Health Programme, Oxford, UK.

## Conflicts of interest

The authors declare they have no conflicts of interest.

## Ethics approval

Ethical approval for the study was granted by the AHC Institutional Review Board, Siem Reap, Cambodia and the Oxford Tropical Research Ethics Committee, United Kingdom.

## Acknowledgments

We thank all members of staff at AHC for help with the study, in particular the directors of the hospital and the laboratory staff.
